# BCAP31 drives TNBC development by modulating ligand-independent EGFR trafficking and spontaneous EGFR phosphorylation

**DOI:** 10.7150/thno.35383

**Published:** 2019-08-21

**Authors:** Wenyan Fu, Hefen Sun, Yang Zhao, Mengting Chen, Xueli Yang, Yang Liu, Wei Jin

**Affiliations:** 1 Department of Breast Surgery, Key Laboratory of Breast Cancer in Shanghai, Fudan University Shanghai Cancer Center, Shanghai 200032, China; 2 Department of Oncology, Shanghai Medical College, Fudan University, Shanghai 200032, China

**Keywords:** TNBC, BCAP31, EGFR, Cancer Development

## Abstract

Identification of novel targets for triple-negative breast cancer (TNBC) is an urgent task as targeted therapies have increased the lifespans of Oestrogen Receptor ^+^/ Progesterone Receptor ^+^ and HER^2+^ cancer patients.

**Methods**: genes involved in protein processing in the endoplasmic reticulum, which have been reported to be key players in cancer, were used in loss-of-function screening to evaluate the oncogenic roles of these genes to identify candidate target genes in TNBC. In vitro and in vivo function assays as well as clinical prognostic analysis were used to study the oncogenic role of the gene. Molecular and cell based assays were further employed to investigate the mechanisms.

**Results**: B Cell Receptor Associated Protein 31 (BCAP31), the expression of which is correlated with early recurrence and poor survival among patients, was identified an oncogene in our assay.* In vitro* studies further suggested that BCAP31 acts as a key oncogene by promoting TNBC development. We also showed that BCAP31 interacts with epidermal growth factor receptor (EGFR) and serves as an inhibitor of ligand-independent EGFR recycling, sustaining EGFR autophosphorylation and activation of downstream signalling.

**Conclusion**: These findings reveal the functional role of BCAP31, an ER-related protein, in EGFR dysregulation and TNBC development.

## Introduction

The incidence of breast cancer, one of the common cancers in women, is increasing worldwide 1. With the implementation of screening, improvements in the local management of early breast cancer and, most importantly, the introduction of adjuvant systemic treatments, the mortality rate in developed countries has declined over the past three decades 2. Additionally, targeted therapeutics have gradually been developed, which has changed and improved the overall treatment of some subgroups of breast cancer, such as HER+ breast cancer. Triple-negative breast cancers (TNBCs) are a subgroup of breast cancers lacking Oestrogen Receptor (OR), Progesterone Receptor (PR), and HER2 expression. TNBC is reported to be associated with shorter progression-free survival (PFS) and overall survival (OS), a poorer prognosis, and a higher risk of relapse than hormone receptor-positive or HER+ breast cancer, even despite optimal treatment 3. As many as 50% of patients who are diagnosed with resectable (stage I-III) TNBC complete tri-modality therapy (surgery ± radiotherapy + adjuvant or neoadjuvant chemotherapy), and as many as 37% of these patients die, with disease recurrence observed in the first 5 years following surgery 4. The standard of care for patients presenting with metastatic TNBC is cytotoxic chemotherapies, such as ixabepilone, capecitabine, anthracyclines and taxanes 5. Moreover, the duration of the response to chemotherapy for TNBC is relatively short. In a retrospective analysis, the mean duration of the response to monotherapy or combination therapy in 111 patients was only 12 weeks after first-line treatment, 9 weeks after second-line treatment, and 4 weeks after third-line treatment 6. Additionally, no specific FDA-approved targeted therapy is currently available to improve TNBC patient outcomes.

The endoplasmic reticulum (ER), which comprises the broad membrane surfaces in the cell extending from the nuclear envelope to the cell periphery, is the first compartment of the secretory pathway 7. Due to a high proliferation rate, cancer cells often encounter defective ATP generation, hypoxia, hypoglycaemia and specific mutations that may disrupt ER homeostasis and trigger ER dysfunction 8. In normal cells, persistent ER stress often energetically eradicates potentially rogue cells by activating pathways that lead to cell death. On the other hand, cancer cells may commandeer the quality control machinery of the ER to produce survival signals, which are required for neoplasm growth, and to ultimately avoid cell death 9. Moreover, a recent report also shows the oncogenic roles of various components of the Unfolded Protein Response (UPR) and Endoplasmic Reticulum-Associated Degradation (ERAD) as potent therapeutic targets that have the ability to modulate the development of cancer cells 10.

The HER receptor tyrosine kinase family includes four members: epidermal growth factor receptor (EGFR, ErbB1/HER1), HER2/neu (ErbB2), HER3 (ErbB3) and HER4 (ErbB4) 11. EGFR is a 170-kDa transmembrane protein with an extracellular domain, a transmembrane domain and an intracellular kinase domain. Ligands of EGFR, such as epidermal growth factor (EGF) and transforming growth factor-alpha (TGF-α), bind to the extracellular domain of EGFR, resulting in homo- or hetero-dimerization/activation of the receptor. This homo- or hetero-dimerization then induces autophosphorylation of the tyrosine kinase domain 12, which serves as a binding site for the recruitment of signal transducers and activators of intracellular substrates. The Ras-Raf mitogen-activated protein kinase pathway and the phosphatidyl inositol 3' kinase and AKT pathways are the major signalling routes for EGFR. These signalling pathways control important biological processes, such as cellular proliferation, angiogenesis and the inhibition of apoptosis 13. Dysregulation of EGFR and the HER receptor family has been shown to be a key process in tumourigenesis and the progression of cancer 14. Therapeutic biomolecules developed to block the HER receptor family, such as the anti-EGFR antibodies panitumumab and cetuximab, the anti-HER2 antibodies trastuzumab and pertuzumab and the EGFR tyrosine kinase inhibitors erlotinib and gefitinib, have been approved by the FDA and have transitioned the approach from bench to bedside 15. Various mechanisms have been reported to underlie the oncogenic activation of EGFR, including transcriptional overexpression, chromosomal translocation, point mutation, and the creation of an autocrine loop 16. However, little is known about the molecules that control protein quality in the ER and their oncogenic roles in cancer development together with EGFR signalling.

Here, we focused on the functional roles of the protein processing components in the ER in TNBCs. We first adopt RNA interference (RNAi) to screen both triple-negative and luminal breast cancer cell lines and identified B Cell Receptor Associated Protein 31 (BCAP31) as a crucial gene in TNBC. Unexpectedly, we identified BCAP31 as an important mediator of ligand-independent EGFR signalling to promote cancer development in TNBCs, and our study more broadly reveals that the collaboration between BCAP31 and EGFR can be exploited for therapeutic interventions.

## Materials and Methods

### Cell lines, plasmids and siRNA

All cancer cell lines obtained from the American Type Culture Collection (ATCC) were routinely assessed for contamination by mycoplasma using Hoechst staining and were consistently found to be negative. During the study period, all cell lines were authenticated twice by morphologic and isoenzyme analyses. The cells were cultured in a humidified incubator in 5% CO_2_ at 37.5°C with DMEM or L15 medium supplemented with 10% foetal bovine serum. All shRNA lentiviral constructs used in the present study were purchased from GeneChem (Shanghai, China). The BCAP31 clones (UniProtKB No. P51572-1) used in this study were all chemically synthesized by GENEWIZ (Shanghai, China). An shRNA-resistant BCAP31 clone with silent mutations against all BCAP31 shRNAs was introduced (Sequence: ATGAGTCTGCAGTGGACTGCAGTTGCCACCTTCCTCTATGCGGAGGTCTTTGTTGTGTTGCTTCTCTGCATTCCCTTCATTTCTCCTAAAAGATGGCAGAAGATTTTCAAGTCCCGGCTGGTGGAGTTGTTAGTGAGCTACGGCAACACCTTCTTCGTGGTTCTCATTGTCATCCTTGTGCTGTTGGTCATCGATGCCGTGCGCGAAATTCGGAAGTATGATGATGTGACGGAAAAGGTGAACCTCCAGAACAATCCCGGGGCCATGGAGCACTTCCACATGAAGCTTTTCCGTGCCCAGAGGAATCTCTACATTGCTGGCTTTTCCTTGCTGCTGTCCTTCCTGCTTAGGAGGCTGGTGACCCTGATCTCGCAGCAGGCCACGCTGCTGGCCTCCAATGAAGCCTTTAAAAAGCAGGCGGAGAGTGCTAGTGAGGCGGCCAAGAAGTACATGGAGGAGAATGACCAGCTCAAGAAGGGAGCTGCTGTTGACGGAGGCAAGTTGGATGTCGGGAATGCTGAGGTGAAGTTGGAGGAAGAGAACAGGAGCCTGAAGGCTGACCTGCAGAAGCTAAAGGACGAGCTGGCCAGCACTAAGCAAAAACTAGAGAAAGCTGAAAACCAGGTTCTGGCCATGCGGAAGCAGTCTGAGGGCCTCACCAAGGAGTACGACCGCTTGCTGGAGGAGCACGCAAAGCTGCAGGCTGCAGTAGATGGTCCCATGGACAAGAAGGAAGAG). For Immunoprecipitation (IP) experiments, c-terminal FLAG Tag was fused to BCAP31. The siRNAs were purchased from GenePharma (Shanghai, China).

### siRNA screening and data analysis

To identify the genes that belong to the protein processing machinery in the ER and are also potential drug targets that are specifically required in TNBCs versus luminal breast cancers, a targeted siRNA library was assembled on the basis of 3 public datasets (KEGG, Protein Atlas, and Drug Bank) and used to identify genes associated with the development of TNBC compared with luminal tumours. Finally, 20 genes were selected.

The gene screen has been described previously. Briefly, the cells were reverse transfected in 96-well plates in triplicate using an siRNA pool library (4 siRNAs/gene) at a final concentration of 100 nmol/L and the appropriate lipid. AlamarBlue (Invitrogen) was added to the wells, and the fluorescence was read using a 96-well fluorometer with excitation at 530 nm and emission at 590 nm. The data were normalized on each plate by dividing the percentage of relative fluorescence units by the average of 2 nontargeting controls. The normalized values from each plate were then averaged, and the Z-scores were calculated using the following formula: (gene value - plate average)/plate SD. The Z-scores from the basal and luminal lines were independently averaged. To reduce the Z-score to a single, comparable value, the luminal Z-score was subtracted from the basal Z-score. A *t* test between the 2 groups was conducted to identify statistically significant hits. Genes with a ΔZ-score less than 0 and a P value greater than 0.05 were considered hits.

### KM plot survival analysis

The 3 hit genes in the present study were entered into the KM plot database (the Kaplan-Meier plotter, KM plot, http://www.kmplot.com/analysis), which can assess the effects of 54,675 genes on survival using 10,293 cancer samples to examine the association between these genes and the 5-year survival rates of the patients. The database includes 5,143 breast, 1,648 ovarian, 2,437 lung and 1,065 gastric cancer samples, with mean follow-up periods of 69, 40, 49 and 33 months, respectively. The primary purpose of this tool is to conduct meta-analysis-based biomarker assessments 17.

### Study population

This study was approved by the Ethics Committee of Fudan University Shanghai Cancer Center (FDUSCC), and each participant signed an informed consent document. A total of 186 breast cancer cases were included in this study. The patients were diagnosed with pathologically invasive ductal breast cancer. The follow-up period was at least 5 years. The diagnoses were verified by two independent pathologists in the Department of Pathology at FDUSCC. The samples were collected from these patients in the Department of Breast Surgery at FDUSCC from August 2001 to March 2006. OS was defined as the interval between surgery and either death or the last observation. relapse-free survival (RFS) was defined as the interval between surgery and recurrence. If recurrence was not diagnosed, then the patients were censored on the date of death or the last follow-up.

### Tissue microarrays, IHC staining, and IHC variable evaluation

Archived formalin-fixed, paraffin wax-embedded samples of carcinomas obtained from the 186 breast cancer patients described above were used to construct tissue microarrays (TMAs; Table 1). Tissue cylinders with diameters of 2 mm were punched from a previously marked tumour area in each block (donor block) and inserted into a recipient paraffin wax block, resulting in a 10 × 12 array. To compare the staining patterns in different areas of the same tumour, tissue samples from all 186 patients were punched twice into the microarray.

A two-step protocol (GTVisionTMIII) was used for the immunohistochemistry of BCAP31. Briefly, TMA sections were washed with phosphate-buffered saline (PBS) after rehydration and then treated with 3% hydrogen peroxide for 10 min to block endogenous peroxidase activity. The antigens were retrieved by boiling the TMAs in citrate buffer (pH 6.0) at 100°C for 5 min. The TMAs were blocked with 10% normal goat serum for 1 hour at room temperature (RT) and incubated in a humid chamber at 4°C overnight with the indicated antibody. Following washes with PBS, all of the TMAs were incubated for 30 min with secondary antibody (GTVisionTMIII Detection System/Mo&Rb) at RT. The sections were counterstained with Gill haematoxylin and mounted after clearing with xylene. TMAs representing duplicate samples from each case were stained and scored semi-quantitatively. Staining was graded based on the staining intensity (0, negative; 1, weak; 2, moderate; and 3, strong) and the percentage of cells stained (1, 0 to <10%; 2, 10 to <50%; and 3, 50 to 100%). Scoring was conducted according to a sum index (SI) of the intensity and the percentage of positive cells as follows: SI, 2, scored as 0; SI, 3, scored as 1; SI, 4, scored as 2; and SI, 5 or 6, scored as 3. If the score was equal to or greater than two, then the tumour was considered to have high expression; otherwise, low expression was determined. Scoring was reviewed in parallel by two experienced breast disease pathologists who were blinded to all clinical data.

### Cell proliferation assay

For cell proliferation assessment, the cancer cells (2000-5000 cells/well) were plated on 96-well plates. AlamarBlue (Invitrogen) was added to the wells after 1-6 days, and the fluorescence was read using a 96-well fluorometer with excitation at 530 nm and emission at 590 nm. The results are expressed in relative fluorescence units compared with the control group.

### Immunostaining, immunoprecipitation, mass spectrometry (MS) and immunoblotting

The experiments were executed as described previously 18. For immunofluorescence staining, cells were plated on cover slides, fixed with 4% paraformaldehyde, permeabilized with 0.3% Triton X-100, and blocked with 1% BSA. The primary antibodies were incubated overnight and subjected to the corresponding fluorescent dye-conjugated secondary antibody for labelling. The localizations were observed, and pictures were captured using a Leica TCS SP2 confocal system (Leica, Germany). For IP, the cells were lysed in buffer (50 mmol/L Tris, pH 7.5, 150 mmol/L NaCl, 1% Nonidet P-40, 1 mmol/L EDTA, a 5-μg/ml aprotinin, pepstatin, and protease inhibitor cocktail tablet and 0.25% deoxycholate). The cell lysates were incubated with 1 μg of primary antibodies or control IgG at 4°C overnight, and then each sample was mixed with 50 μl of protein-G conjugated beads (Roche Molecular Biochemicals). Lysis buffer was used to wash the beads, and the immunoprecipitated protein complexes were separated on SDS-PAGE gels followed by silver staining or analysis by western blotting. For mass spectrometry (MS), the bands were extracted from the gel and analysed by liquid chromatography-tandem mass spectrometry (LC-MS/MS) sequencing as described previously 19. Briefly, the proteins were digested in-gel and extracted. The extracted peptides were mixed with matrix and then spotted onto a sample plate. Time-of-flying (TOF, ABI 4700 protein analyser, ABI) was used to identify the mass of the peptides. The data from MS and LC-MS/MS were searched against the Swiss-Prot database (*Homo sapiens*). All proteins deemed to be high-confidence interactors of BCAP31 were identified by at least two unique peptides. For signalling assays, cells with different treatments were incubated in serum-free medium for 1 hour at RT and then treated with EGF (0.5 nmol/L) for 15 min as indicated. After washing, the cells were lysed, and the lysates were subjected to SDS-PAGE and immunoblot analyses with antibodies against EGFR, phospho-EGFR-Tyr1068, phospho-EGFR-Tyr845, AKT, phospho-AKT-Ser473, p44/42 MAPK, and phospho-p44/42 ERK1/2 Thr202/Tyr204 (all from Cell Signaling Technology). All the ELISA kits were provided by Cell Signaling Technology, and the catalogue numbers are #7189 (Tyr845 of pEGFR), #7134 (Ser473 of pAKT), #7240 (Tyr1068 of pEGFR), and #7177 (Thr202/Tyr204 of pERK). All assays were performed independently three times.

### Migration assay

A modified two-chamber migration assay (8-mm pore size, BD Biosciences, Bedford, MA) was adopted to assess cell mobility according to the manufacturer's instructions as described previously 20. Approximately 2 × 10^4^ cells were plated into the upper chamber, and the cells were then allowed to migrate into the lower chamber for 18-24 hours. The cells at the bottom of the membrane were fixed and stained with methanol 20%/crystal violet 0.2%, while the cells in the upper chamber were removed using cotton swabs. The data are presented as the means ±standard error of the mean (SEM).

### Colony formation assay

For the colony formation assay, the cells were seeded onto 6-well plates or 3.5-cm dishes. Colonies were allowed to form in an incubator at 37°C and 5% CO_2_ for 10 days. At the end of the incubation period, the clones were fixed and stained with 0.5% crystal violet, and colonies larger than 50 μM in diameter were counted.

### *In vitro* invasion assay

The invasion assay using transwell cell culture chambers (8-μM pore size polycarbonate membrane, Costar) was performed as previously described 21. In brief, 100 μl of cell suspension at 1 × 10^6^ cells/ml in DMEM supplemented with 0.5% foetal bovine serum was loaded into the upper chamber, and the lower chamber was loaded with 600 μl of DMEM with 10% foetal bovine serum. The membranes were precoated with Matrigel (BD Pharmingen). The chamber plates were incubated at 37°C for 24 hours. Then, the filter was fixed in 4% paraformaldehyde and stained with haematoxylin (Sigma). The cells on the upper side of the filter were wiped off with a cotton swab, and the cells that had passed through the membrane were counted in 10 randomly selected microscopic fields. Each assay was performed in triplicate.

### In vivo assay

The animal experiment protocols were approved by the Animal Ethics Committee of Shanghai Medical College, Fudan University. All animals were housed in a specific pathogen-free room with a 12-hr light/dark schedule at 25°C±1°C. Six-week-old female nude mice (BALB/c) were randomly divided into the indicated groups before inoculation, and a double-blinded evaluation was performed when measuring tumour volumes with callipers at least once a week for the duration of the study. Tumours were measured with digital callipers, and tumour volumes were calculated by the formula: volume = length × (width)^2^/2. The mice were sacrificed at the end of the study, and their tumours and all organs were removed and examined for metastatic nodules. In the study, we found metastatic nodules only in the liver, and whole lungs were fixed with paraformaldehyde (4%) before dehydration and embedding in paraffin. Paraffin sections were stained with haematoxylin & eosin staining (H&E) according to standard protocols, and metastatic nodules in the livers were counted with a microscopic count assay.

### Flow cytometry analysis

The cells were detached using EDTA, resuspended in growth medium and counted. The cells (0.5 × 10^6^) were resuspended in 100 μl of PBS after washing. The cells were stained with FITC-labelled anti-EGFR antibody on ice for 60 min in the dark prior to washing and analysis. At least 1 × 10^4^ cells per sample were analysed with flow cytometry.

### Phosphorylation assays

To analyse pEGFR and pAKT *in vivo*, the tumour samples were homogenized in cell lysis buffer (CST; Cat# 9803). The ratios of pEGFR to total EGFR, pAKT to total AKT and pERK to total ERK were determined by ELISA.

### RNA isolation and quantitative RT-PCR

The RNeasy Mini Kit (Qiagen) was used to isolate total RNA according to the manufacturer's specifications. Real-time quantitative PCR was performed on an ABI PRISM 7900HT and analysed using SDSv2.3 (Applied Biosystems). β-Actin was used as an endogenous control to normalize expression. Quantitative real-time PCR was performed using a commercially available TaqMan EGFR probe (EGFR, Hs01076090_m1).

### Biotinylation assay

Cancer cells were washed with PBS and then surface biotinylated by incubation with 1 mg/ml biotin (Thermo) for 30 min, washed, and harvested for purification of biotinylated proteins using EZview Red Streptavidin Affinity Gel (Sigma-Aldrich). Whole-cell lysates and purified biotinylated proteins were analysed by immunoblotting.

### Receptor recycling assays

Cells were plated and transfected with the indicated shRNAs. After 24 hours, EGFR recycling assays were performed as previously reported 22. Briefly, the cells were surface-labelled with Sulfo-NHS-SS-biotin at 4°C, and ligand-induced internalization was initiated by incubating the cells for 10 min with EGF at 37°C to allow EGFR to accumulate in early endosomes. The remaining surface biotin was stripped by three 10-min incubations in MESNA solution. The cells were then rewarmed to 37°C in serum-free medium without ligand for either 5, 15 or 30 min to allow recycling of internalized biotinylated receptors. The membrane proteins were extracted and analysed for recycled receptors. For ligand-independent recycling, the cells were surface-labelled with Sulfo-NHS-SS-biotin at 4°C and then cultured at 37°C for 4 hours to allow EGFR internalization, and subsequently, the surface biotin was stripped. The cells were then rewarmed to 37°C for 24, 48 and 72 hours before analysis. Biotinylated EGFR was then assessed by capture-ELISA.

### Capture-ELISA

Capture-ELISA were performed as previously reported 23. Briefly, 96-well plates (Life Technologies) were coated overnight with 5 μg/ml anti-EGFR antibodies at 4°C and blocked in PBS containing 0.05% Tween-20 (PBS-T) with 5% BSA for 1 hour at RT. EGFR was captured by overnight incubation of 50 μl of cell lysate at 4°C. The wells were washed and incubated with streptavidin-conjugated horseradish peroxidase (Amersham) in PBS-T containing 1% BSA for 1 hour at 4°C. Following further washing, biotinylated EGFR was detected by a chromogenic reaction with ortho-phenylenediamine.

### Statistical analyses

Statistical analyses were performed using SPSS 16.0 software (SPSS) and PRISM 5.0 (GraphPad Software Inc.). Comparisons of quantitative data between two groups were analysed using Student's *t* test (two-tailed; *P* < 0.05 was considered significant). The χ^2^ test was used to compare qualitative variables. The cumulative survival time was calculated using the Kaplan-Meier method, which was analysed using the log-rank test. Univariate and multivariate analyses were based on the Cox proportional hazards regression model. *P* values < 0.05 were considered significant.

## Results

### BCAP31 is overexpressed in triple-negative breast cancer and is essential for tumour development

**Figure 1:**
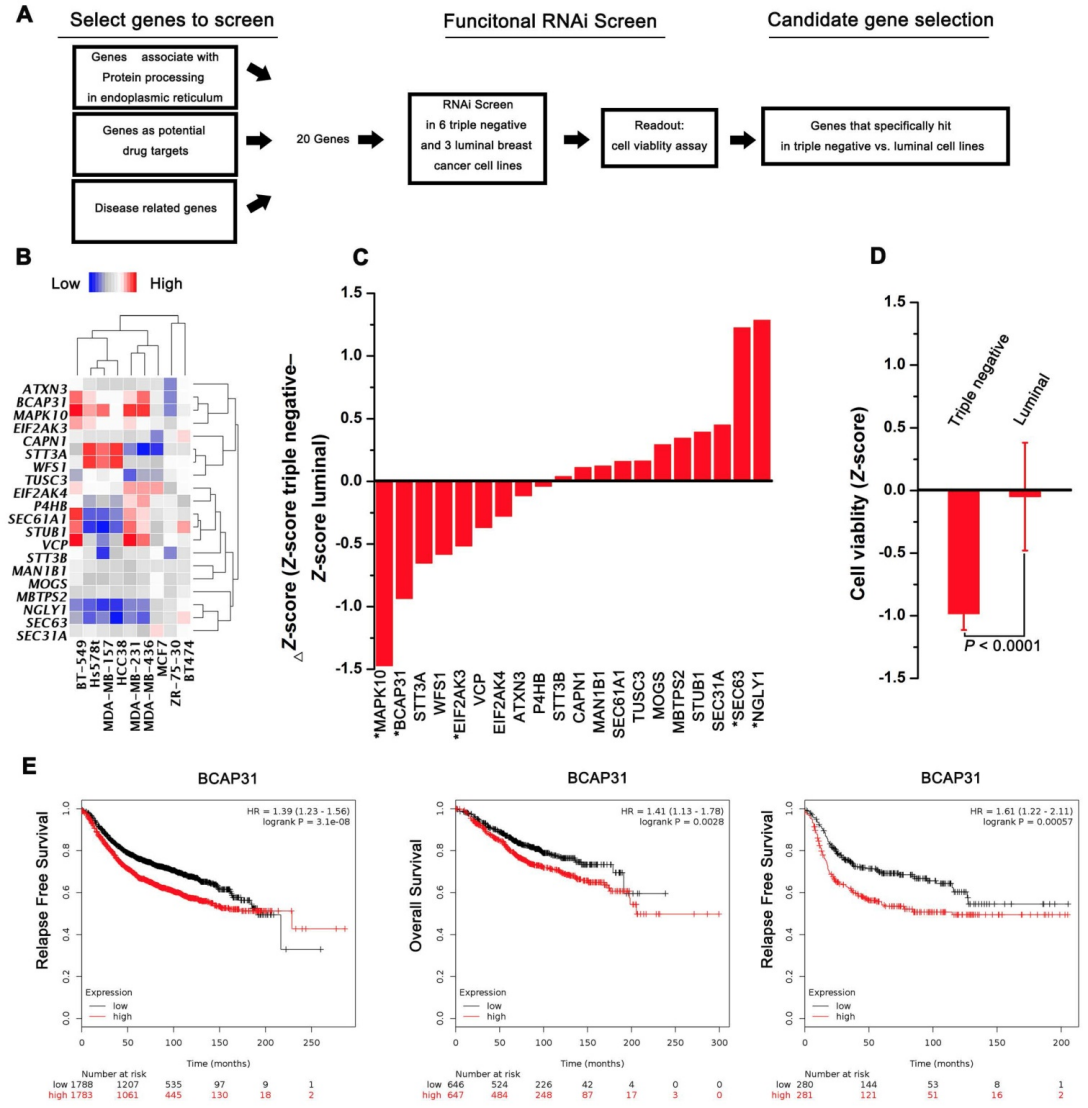
** RNAi screening and KMplot strategy to identify regulators of basal-like breast cancer. (A)** Schematic highlighting the criteria for gene selection and the experimental setup of the RNAi screen. **(B)** Heat map representing the expression of selected transcripts in different cells as determined by qPCR analysis. **(C)** Column showing the average Z-score of triple-negative cell lines minus the average Z-score of luminal cell lines (ΔZ-score) for all the genes included in the RNAi screen. A negative value indicates that the siRNA decreased cell proliferation more in the triple-negative cell lines. * P < 0.05 comparing triple-negative and luminal Z-scores. **(D)** The average triple-negative and luminal Z-scores for all cell lines for BCAP31. The P value indicates a statistically significant difference between triple-negative and luminal lines. Error bars represent the SD. **(E)** Kaplan-Meier survival curves showed poorer RFS and OS with high BCAP31 expression than those with low BCAP31 expression in breast cancer, left two panels, and in basal-like breast cancer, right panel.

A functional RNAi screen was first conducted to identify genes that are selectively required for cell proliferation in TNBC (Figure 1A). Candidate genes for the screen were selected on the basis of their associations with protein processing in the ER, their potential as drug targets, and their correlations with disease based on 3 public datasets (KEGG, Protein Atlas, and Drug Bank). Twenty candidate genes were selected for screening across 6 triple-negative and 3 luminal cell lines, and cell proliferation was quantified at 4 days post transfection. The expression of selected transcripts in different cells was first determined by qPCR analysis (Figure 1B). The average Z-score was calculated for genes with a differential cell proliferation effect between the triple-negative and luminal breast cancer cell lines. Our data revealed 3 genes (MAPK10, BCAP31, and EIF2Ak3) that met our significance criteria of scoring at a ΔZ-score level (triple-negative cell line Z-score - luminal cell line Z-score) (Figure 1C-D). To further validate the 3 genes identified, the KM plot was used to analyse the survival potential of patients with upregulated genes. Following gene upload, based on 336-3,571 candidate patients, we found that high BCAP31 expression was significantly associated with low relapse-free survival (RFS) in a cohort of 3,571 patients and with low OS in a cohort of 1,293 patients. Moreover, high BCAP31 expression was also significantly associated with low RFS in a cohort of 561 basal-like breast cancer patients (Figure 1E). However, different MAPK10 and EIF2AK3 expression levels did not exhibit a significant association with clinical outcomes (Figure S1). Therefore, BCAP31 was selected for further evaluation.

### BCAP31 expression is associated with breast cancer patient outcomes

**Figure 2:**
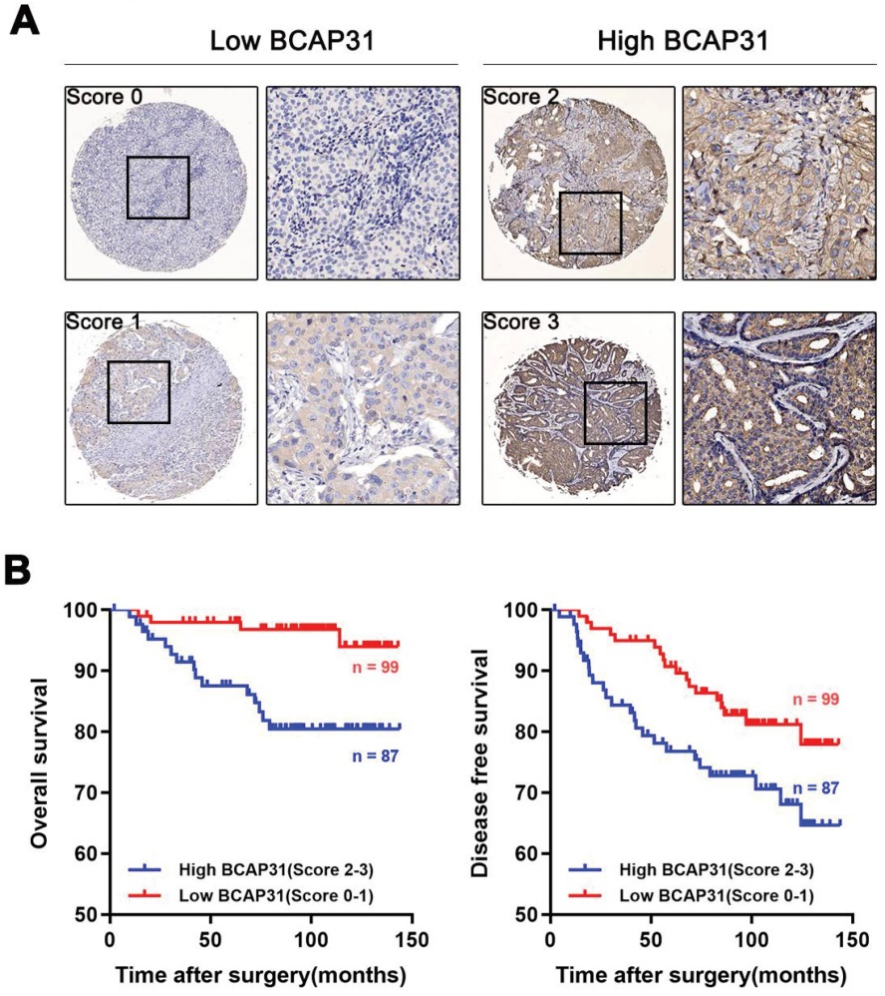
** Upregulation of BCAP31 correlates with a poor prognosis for human breast cancer. (A)** The scores indicate BCAP31 levels in representative tumour tissues. The scores were calculated by the intensity and percentage of stained cells as described in the Methods. **(B)** Patients with high BCAP31 expression (score of 2-3) have poorer overall survival and a higher probability of recurrence than patients with low BCAP31 expression (score of 0-1).

To further investigate the association of breast cancer development with the expression and prognostic value of BCAP31, an IHC study of BCAP31 in 186 breast cancer-based tissue microarrays with comparable clinicopathological features and complete follow-up data was performed (Table 1). The cases were divided into low (score of 0-1) or high (score of 2-3) BCAP31 expression groups according to the immunostaining scores (Figure 2A). Our data showed that high BCAP31 expression was significantly associated with the molecular subtype of breast cancer (high BCAP31 expression in TNBC, Table 1), reduced OS (p = 0.0019), and reduced disease-free survival (DFS, p = 0.0411) in 186 breast cancer patients (Figure 2B). Cox proportional hazards regression analyses also showed that overexpression of BCAP31 was an independent prognostic predictor for both OS (hazard ratio [HR] = 0.2026, p = 0.0053) and DFS (HR = 0.5170, p = 0.0370; Supplementary Table S1) in 186 breast cancer patients (Supplementary Table S1). These results indicate that BCAP31 overexpression is significantly associated with the poor prognosis of breast cancer patients.

### BCAP31 plays an oncogenic role in TNBC cells

The above results suggest that BCAP31 may play an important role in breast cancer development. Subsequently, we assessed the function of BCAP31 in breast cancer cells using both* in vitro* and *in vivo* assays.

**Figure 3:**
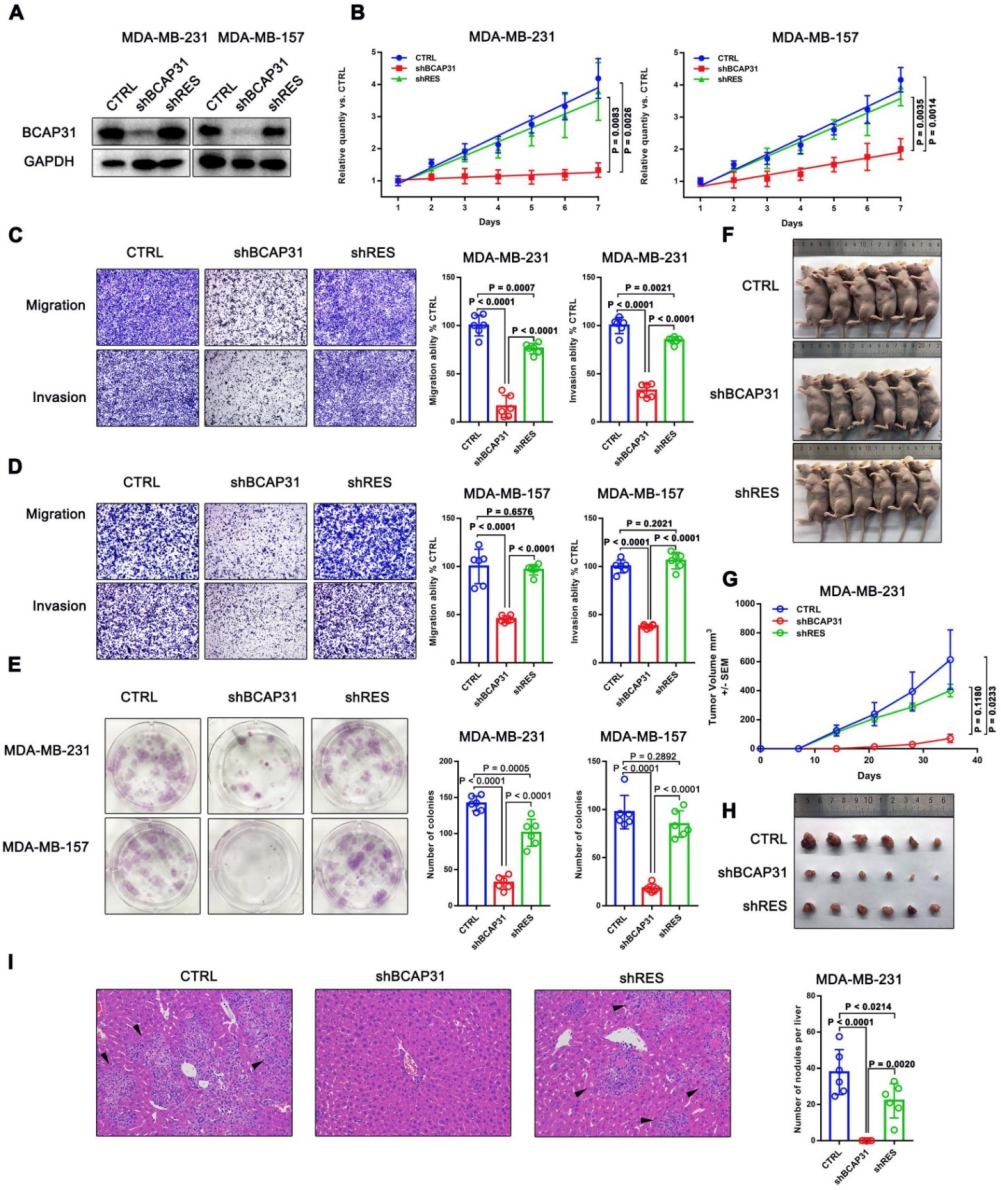
** The roles of BCAP31 in promoting breast cancer development. (A)** Confirmation of BCAP31 knockdown (KD, shBCAP31) and re-expression (shRES) in TNBC cell lines. **(B-E)** The effects of BCAP31 knockdown or re-expression on the *in vitro* proliferation **(B)**, **(C and D)** migration, invasion and **(E)** colony formation of HCC cells. Error bars indicate the means ± SEM. **(F)** *In vivo* tumour growth in MDA-MB231 shBCAP31 mice was significantly inhibited compared with control mice. Representative images of tumour-bearing mice are shown. **(G)** The dynamic change in tumour volume in subcutaneous models is shown. Error bars indicate the means ± SEM. Knockdown of BCAP31 significantly decreased tumour growth **(H)** and spontaneous liver metastasis **(I)** in MDA-MB-231 xenograft nude mice models. Representative images of H&E-stained liver tissues from the xenograft groups. Arrowheads indicate metastatic nodules in the liver.

We first investigated the endogenous BCAP31 levels of different breast cancer cell lines and found a notable increase in BCAP31 expression levels in TNBC cell lines, such as MDA-MB-231 and MDA-MB-157 cells, which have higher invasive and metastatic capabilities than luminal cell lines, i.e., ZR-75-1, BT474, and MCF-7 cells, which have lower metastatic potential (Figure S2). Since our above results indicated an oncogenic role for BCAP31 in TNBC cells, we then used TNBC cell lines in further experiments. We modulated BCAP31 expression levels via lentivirus-mediated BCAP31-specific short hairpin (sh) RNAs in MDA-MB-231 and MDA-MB-157 cells to evaluate the effects of BCAP31 on *in vitro* cell proliferation, migration, and invasion. Three BCAP31-specific shRNAs were generated to silence endogenous BCAP31 expression (shBCAP31) in breast cancer cells. shBCAP31#2, which was adopted for further study, showed the most significant knockdown (KD) effect in our assays (Figure 3A and Figure S3). Although BCAP31 is an ER regulator, our data show that neither knock-out nor re-expression of BCAP31 affects the expression of GAPDH or β-Actin (Figure S4). BCAP31-KD induced by shBCAP31 resulted in a notable inhibitory effect on the *in vitro* proliferation of both MDA-MB-231 and MDA-MB-157 cells (Figure 3B). We next sought to exclude the possibility of off-target effects via the BCAP31-KD method. Reintroducing BCAP31 with engineered cDNA that was not sensitive to shRNA (shRES) into BCAP31-KD cells returned the repressed proliferation to almost normal levels in both BCAP31-KD cell lines (Figure 3B).

In migration, invasion and colony formation assays, BCAP31 downregulation also induced potent suppression of TNBC cells, while re-introduction of BCAP31 returned cell function to nearly normal levels (Figure 3C-E). To confirm these findings, the effects of BCAP31 expression on the *in vivo* tumour growth and metastasis of TNBC cells were further examined. Tumour growth was monitored in subcutaneous implantation nude mice models. Our data showed that the nude mice injected with shBCAP31-transfected MDA-MB-231 cells (-shBCAP31) had much smaller tumour sizes than those injected with BCAP31 cells transfected with non-target shRNA control (CTRL) or cells with re-expressed shRNA-resistant BCAP31 (Figure 3F-H). At the end of the study, the mice were sacrificed, and all organs were removed to search for metastases. Only liver metastases were found. Consecutive sections were then taken from every liver tissue block and stained with H&E. The number of liver metastases was evaluated and calculated independently by two researchers. Interestingly, we found no significant liver metastasis foci in any subcutaneous implantation model of the shBCAP31 group, while the mean number of metastases per liver in the control group and shRES group were 57.5 and 32.9, respectively (p < 0.0001, Figure 3I).

### BCAP31 interacts with EGFR according to interactome analyses and correlates with ligand-independent EGFR/AKT pathway activation

**Figure 4:**
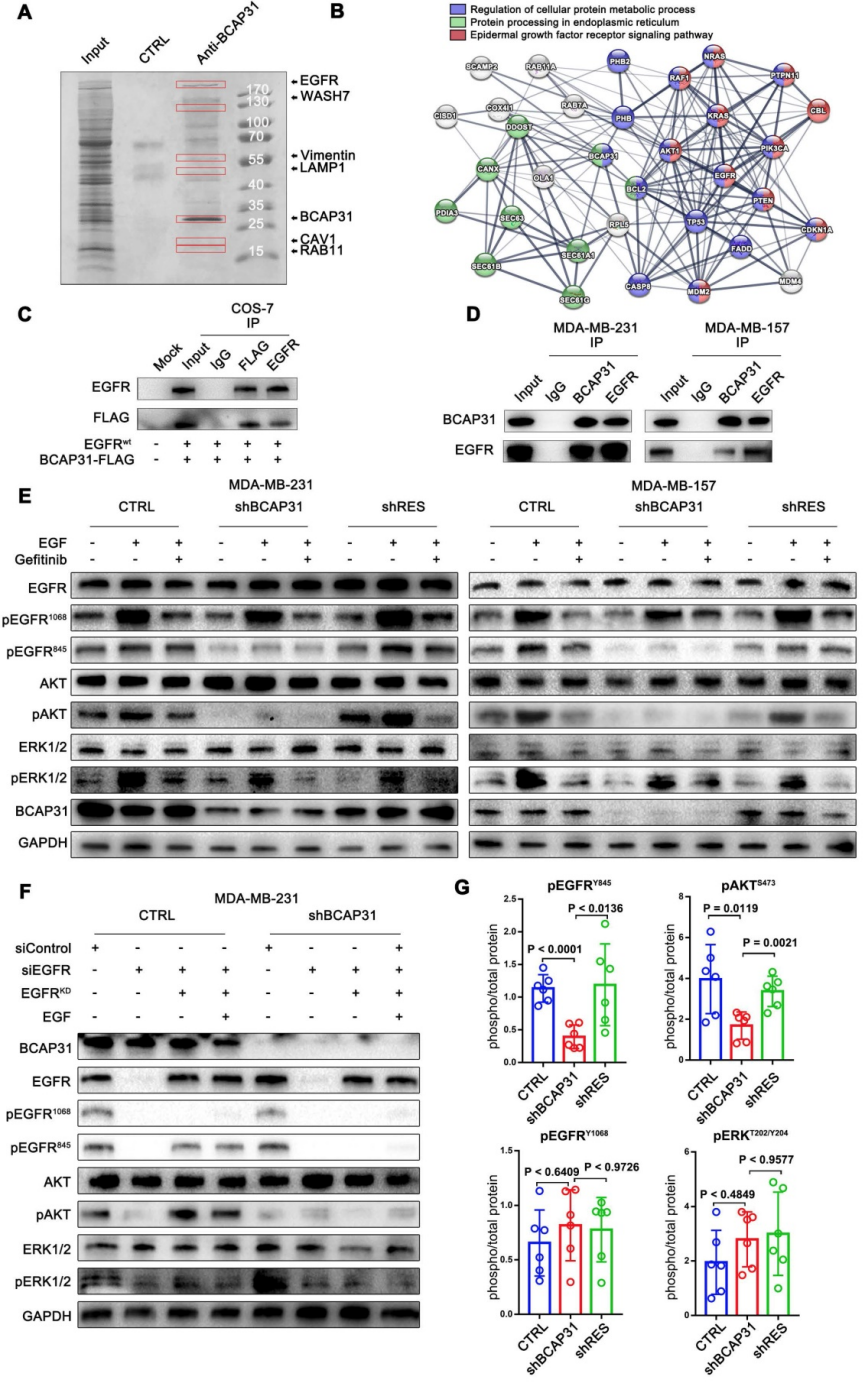
** BCAP31 interacts with EGFR and facilitates ligand-independent EGFR/AKT pathway activation. (A)** Mass spectrometry (MS) analysis of BCAP31-associated proteins. The purified protein complex was resolved on SDS-PAGE, followed by silver staining; then, the bands were retrieved and analysed by MS.** (B)** Analyses of the identified BCAP31 interactors. The diagram depicts BCAP31 interactors as detected by MS. The network was built based on the interaction network of EGFR-associated signalling and trafficking processes in the KEGG database overlaid with MS data. **(C and D)** The interaction between exogenous and endogenous BCAP31 and EGFR. Co-IP assay and immunoblot analyses evaluating the BCAP31-EGFR interaction in COS-7 cells and TNBC cells. **(E)** Downregulation of BCAP31 significantly attenuates ligand-independent signalling. TNBC cells stably expressed shBCAP31, control shRNA, or shRES when treated with EGF or EGF combined with gefitinib. IB examinations of phosphorylated EGFR and downstream signalling are shown.** (F)** Re-expression of the K721A kinase-dead (EGFR^KD^) EGFR rescues the ligand-independent phosphorylation of EGFR at Tyr 845 and downstream AKT signalling in EGFR-knockdown MDA-MB-231 cells expressing control shRNA but not shBCAP31. SiRNA-resistant EGFR-KD was expressed in control or shBCAP31-MDA-MB-231 cells by lentivirus-mediated infection. Endogenous EGFR was knocked down by siRNA, and whole-cell lysate was harvested for western blot analysis. **(G)** Tumours of MDA-MB-231 tumour xenografts were harvested, homogenized, and analysed for pEGFR (Tyr1068, Tyr845), pAKT (Ser473), total EGFR and total AKT by ELISA. Statistical significance was determined using Dunnett's test.

To further elucidate the molecular mechanisms of BCAP31 in promoting breast cancer development, binding partners of BCAP31 were purified using immune affinity purification 24 and resolved using liquid chromatography-tandem mass spectrometry (LC-MS/MS) (Figure 4A). Several proteins were identified (Table S2). Analysis of the MS data indicated that the oncoprotein EGFR potentially interacted with BCAP31 (Figure 4B). Both exogenous and endogenous interactions of BCAP31 with EGFR were confirmed by co-immunoprecipitation (co-IP) in COS-7 cells as well as in MDA-MB-231 and MDA-MB-157 cells (Figure 4C-D). As dysregulation of EGFR is intimately associated with the oncogenesis and metastasis of different cancers 25, we hypothesized that BCAP31 promotes cancer development through regulation of the EGFR signalling pathway. In our experiments, western blotting confirmed that 10-min stimulation with 100 ng/ml EGF stimulated strong EGFR Y1068 and Y845 phosphorylation and downstream MAPK and AKT phosphorylation and activation.

Interestingly, basal and EGF-dependent EGFR Y845 phosphorylation and downstream AKT activation, but not EGFR Y1068 phosphorylation and downstream MAPK activation, were significantly decreased when BCAP31 was downregulated by shBCAP31 and could be rescued by re-expression of shRNA-resistant BCAP31 (shRES) in both MDA-MB-231 and MDA-MB-157 cells (Figure 4E). Moreover, adding both EGF and the EGFR kinase blocker gefitinib markedly inhibited ligand-induced EGFR Y1068 phosphorylation and downstream MAPK activation independent of the BCAP31 expression level, while the phosphorylation of EGFR Y845 was observed to be relevant to ligands and inhibitors but was strongly associated with BCAP31 expression.

To further support our hypothesis, siRNA-resistant K721A kinase-dead (EGFR^KD^) EGFR 26 was re-expressed in MDA-MB-231 cells in which endogenous EGFR was knocked down, and EGFR phosphorylation and downstream signalling were assessed. Re-expressed EGFR^KD^ is autophosphorylated and activates AKT signalling through EGFR Y845 phosphorylation; however, when stimulated with EGF, EGFR-KD does not autophosphorylate or activate ERK signalling or EGFR Y1068 (Figure 4G). However, re-expression of EGFR^KD^ rescued EGFR expression in EGFR-KD and BCAP31-KD cells, but neither EGFR Y845 nor Y1068 sites showed autophosphorylation or downstream activation (Figure 4G), indicating that the role of BCAP31 in EGFR signalling is kinase-independent and Y845-dependent. We also examined whether BCAP31-KD inhibited EGFR signalling *in vivo*. The tumour lysates from Figure 3 were subjected to ELISA (Figure 4G). BCAP31-KD (Figure 4F) significantly reduced the level of pEGFR (Tyr845) in the tumours (p < 0.0001, Dunnett's test). Similarly, pAKT (Ser473) was significantly reduced in BCAP31-KD tumours (p = 0.0119), whereas pEGFR 1068 and MAPK did not show significant variance. Together, these data demonstrate that BCAP31 inhibits the EGFR Y845/AKT pathway *in vivo*.

### BCAP31 inhibits ligand-independent spontaneous EGFR phosphorylation

**Figure 5:**
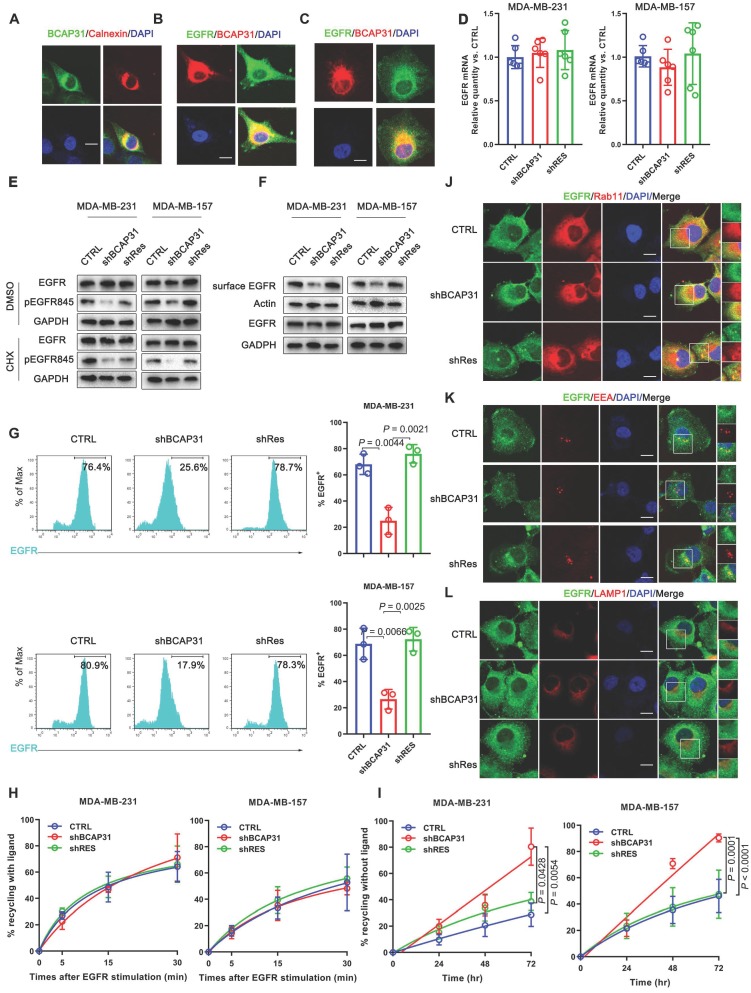
** BCAP31 inhibits EGFR auto-recycling. (A)** MDA-MB-231 cells were fixed for costaining of endogenous BCAP31 (green) and Calnexin (red). **(B)** MDA-MB-231 cells were fixed for costaining of endogenous EGFR (green) and BCAP31 (red). **(C)** Transfected COS-7 cells were fixed for costaining of exogenous EGFR (green) and BCAP31 (red). **(D)** (A) EGFR mRNA expression was determined by qPCR analysis. **(E)** Cycloheximide (CHX) did not suppress EGFR degradation in BCAP31 KD cells. An EGFR degradation assay was performed in the absence or presence of CHX (50 μg/ml). **(F)** TNBC cells were surface biotinylated, and surface and total EGFR levels were analysed by western blotting. **(G)** Representative flow cytometric histograms of TNBC cells examined for the expression of cell surface EGFR with or without BCAP31 knockdown. The experiment was repeated three times independently with similar results. **(H)** The ligand-induced recycling of EGFR was determined in TNBC cells stably expressing shNT, shBCAP31, and shRES vectors as indicated. Error bars indicate the means ± SEM. **(I)** The ligand-independent recycling of EGFR was determined in TNBC cells. MDA-MB-231 cells were fixed for costaining of endogenous EGFR (green) and RAB11 **(J)**, EEA **(K)**, and LAMP1 **(L)** (red). Bar, 10 μm

To understand how BCAP31, a triple transmembrane protein mainly anchored in the ER (Figure 5A), functionally drives breast cancer development in cooperation with the membrane protein EGFR in a ligand-independent manner, we observed the intracellular distribution of BCAP31 and EGFR in the absence of ligand by confocal microscopy (Figure 5B). Immunofluorescence staining showed the large-area co-localization of endogenous EGFR with BCAP31 in MDA-MB-231 cells, which was also observed upon ectopic expression of EGFR and BCAP31 in COS-7 cells (Figure 5C). We then analysed EGFR mRNA levels and found no significant differences between control, BCAP31-KD and shRES cells among both MDA-MB-231 cells and MDA-MB-157 cells (Figure 5D). The treatment of cells with the protein synthesis inhibitor cycloheximide (CHX) did not alter the kinetics of EGFR degradation in BCAP31-downregulated cells (Figure 5E).

To further investigate the BCAP31-mediated regulation of EGFR, FACS analysis and western blotting were used to quantify the amount of cell-surface EGFR. Unexpectedly, we found that although the total EGFR level was not affected in BCAP31-KD cells, the level of EGFR at the cell surface was reduced in TNBC cells (Figure 5F-G), indicating that BCAP31 may mediate EGFR intracellular trafficking. Intracellular trafficking, especially the endocytosis and recycling of cell-surface EGFR proteins, has recently attracted considerable interest in cancer development and regulation research 27. BCAP31 did not affect ligand-induced EGFR activation and signalling, indicating that BCAP31 does not participate in EGFR trafficking under the condition of ligand binding, which was further confirmed with labelled EGFR tracing experiments using EGF stimulation.

We found no significant difference in the recycling rate of EGFR between shBCAP31- and control shRNA (shNT)-treated cells (Figure 5H) following EGF stimulation; however, KD of BCAP31 expression resulted in an increased recycling rate of internalized EGFR at the plasma membrane (Figure 5I) in a 72-hour experiment without ligand. Moreover, compared with control cells, co-localization analysis via confocal microscopy also showed that a substantial amount of EGFR predominantly co-localized with RAB11-positive recycling vesicles in BCAP31-KD cells (Figure 5J and Figure S5A), but co-localization with EEA-positive endocytic vesicles was notably decreased (Figure 5K and Figure S5B). We also found no significant difference in co-localization between shBCAP31 and shNT-treated cells using the degradation marker Lamp1 (Figure 5L and Figure S5C). These data indicated that BCAP31-KD caused re-distribution of EGFR from the cell surface and EEA-positive vesicles to RAB11-positive vesicles, which also caused a decrease in EGFR Y845 and AKT phosphorylation. In summary, these data support that BCAP31 is involved in the regulation of ligand-independent EGFR recycling.

### RAB11 is involved in BCAP31-mediated EGFR recycling inhibition

**Figure 6:**
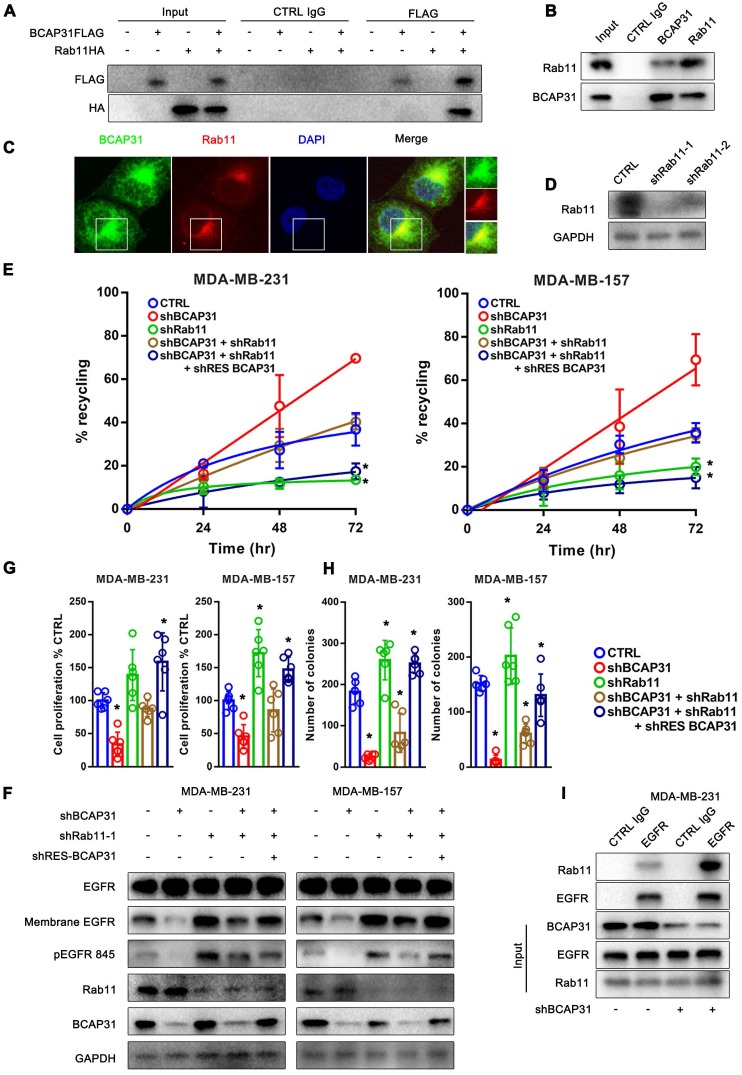
** RAB11 is required for BCAP31-mediated EGFR recycling inhibition and cancer development. (A)** BCAP31 interacts with RAB11. The indicated constructs (BCAP31-FLAG and RAB11-HA) were transiently expressed in COS-7 cells, and the whole-cell lysates were immunoprecipitated (IP) with the indicated antibody. **(B)** *In vitro* interaction between BCAP31 and RAB11. Whole-cell lysates from MDA-MB-231 cells were prepared, and IP and IB were performed with antibodies as indicated. **(C)** MDA-MB-231 cells were fixed for costaining of endogenous BCAP31 (green) and RAB11 (red). **(D)** Confirmation of RAB11 knockdown using western blotting. **(E-H)** The effects of KD of BCAP31 and RAB11 on the ligand-independent recycling **(E)**, ligand-independent signalling** (F)**, cell proliferation **(G)**, and colony formation **(H)** of TNBC cells stably expressing the indicated shRNA vectors. The fold differences represent the mean of the experimental group compared with that of the controls. Error bars indicate the means ± SEM. * p < 0.05 compared with the CTRL. **(I)** Immunoprecipitation of endogenous EGFR from control or BCAP31-knockdown MDA-MB-231 cells. Bar, 10 μm.

The well-known recycling compartment-localized GTPase RAB11 is involved in the recycling of Receptor Tyrosine Kinases (RTKs) to the plasma membrane and has been proven to play a significant role in cancer development 28. Previous studies have reported that the incorporation of RAB11 into a complex with EGFR enhanced EGFR recycling 29, 30. Moreover, our mass spectrometry data suggested a direct interaction between RAB11 and BCAP31 (Supplementary Table S2). Therefore, we assessed whether RAB11 is involved in BCAP31-related EGFR recycling regulation and further investigated the related mechanism. Reciprocal IP assays showed that BCAP31 co-immunoprecipitated with RAB11A in both exogenous and endogenous manners in COS-7 cells and MDA-MB-231 cells (Figure 6A-B). In addition, we found that BCAP31 and RAB11 co-localized inside the cells (Figure 6C). These observations prompted us to investigate whether BCAP31-mediated EGFR endosomal trafficking occurs through its interaction with RAB11.

Consistent with this notion, our data showed that KD of RAB11 expression resulted in a reduced EGFR recycling rate (Figure 6D-E) but an increased membrane EGFR level and EGFR Y845 activation (Figure 6F), accompanied by increased cell proliferation and colony formation *in vitro* (Figure 6G-H) in contrast to the effect of BCAP31-KD.

Moreover, the recycling of EGFR was completely dependent on RAB11 as receptor recycling was blocked by KD of RAB11, even with the re-introduction of BCAP31 into BCAP31-KD cells (Figure 6E). However, KD of BCAP31 suppressed the inhibitory role of EGFR Y845 signalling and the cell viability of MDA-MB-231 shRAB11 cells (Figure 6F). Thus, these results support the notion that endogenous levels of BCAP31 drive tumour cell development through inhibition of the RAB11-dependent recycling mechanism. Consistently, the enhanced interaction of RAB11 with EGFR was found in the absence of BCAP31 as detected by a co-IP assay (Figure 6I), suggesting a role for endogenous BCAP31 in the inhibition of the physical RAB11-EGFR complex to facilitate the return of these receptors to the cell surface and therefore drive EGFR downstream signalling and tumour development.

## Discussion

EGFR overexpression and hyperactivation by genetic alterations have been proven to contribute to malignant transformation 31. Here, we showed that BCAP31 encodes an ER- and vesicle-associated protein that plays a critical role in TNBC development. BCAP31 has been previously suggested to be a key component and quality-control checkpoint in the ERAD pathway, which regulates the fate of integral ER membrane proteins as a molecular chaperone and a quality-control factor by binding to translocon-associated components (SEC61β and TRAM1) 32-35. Together with the ER chaperone BiP, BCAP31 promotes the ER-to-cytosol dislocation of non-enveloped viruses 36. BCAP31 is also an important factor of apoptosis because this protein interacts with Bcl-2/Bcl-xL and procaspase-8L 37 and is also associated with complex crosstalk between the ER and the mitochondrial outer membrane 38, 39. Because of the limited evidence that quality-control checkpoints at the ER regulate cancer development, we focused on the functional roles and related mechanisms of BCAP31 in driving cancer development.

Studies have confirmed that EGFR is a critical oncogenic unit in breast cancer cell lines 40. However, the initial effect of EGFR inhibitors to block EGFR ligands in breast cancer treatment is disappointing 41. Moreover, previous studies have demonstrated that autocatalysis can lead to amplified self-activation of EGFR in the absence of a cognate ligand 42, 43.

Because the Grb2 (Y1068) docking site of EGFR is the main phosphorylation site induced by ligand stimulation 44 and a recent report showed that autocatalytic phosphorylation of tyrosine 845 on unliganded EGFR is regulated by vesicular recycling through perinuclear areas 45, we hypothesized that BCAP31 may be involved in the ligand-independent spontaneous activation of EGFR at the Y845 site. Mechanistically, we provide direct evidence that BCAP31 may promote TNBC through its regulation of EGFR via oncogenic ligand-independent phosphorylation of the conserved regulatory tyrosine Y845 in the activation loop of the EGFR kinase domain 46, resulting in activation of AKT signalling and cancer development in TNBC (Figure 4). We established an association between BCAP31 binding with EGFR and subsequent phosphorylation of EGFR Y845, which in turn causes activation of AKT signalling and correlates with the inhibition of EGFR recycling related to RAB11 (Figure 5).

EGFR activation is strictly controlled in normal cells. The auto-inhibitory interactions of EGFR include local intrinsic disorder of the αC helix in the N-lobe of the kinase domain 47 and receptor-membrane interactions as well as the tethered conformation of the extracellular domain 48. Mutation-induced increases in receptor expression levels or the loss of auto-inhibitory interactions successively enhance basal phosphorylation and maintain the activation status of EGFR 49.

Ligand binding leads to receptor dimerization 50 and the formation of an asymmetric dimer of the intracellular kinase domains 51. This activity triggers trans-phosphorylation of regulatory and signalling tyrosine residues in the intracellular part of the receptor and subsequent recruitment of adaptor proteins that contain Src homology 2 domains (SH2) or phosphotyrosine-binding domains (PTB), such as c-Cbl (Y1045) or Grb2 (Y1068 and Y1086) 52. Once bound, receptor-ligand complexes are endocytosed into clathrin-coated vesicles (CCVs), which further fuse with early endosomes (EEs) 53, mature into late endosomes (LEs) in the perinuclear area, and eventually fuse with lysosomes in which the complexes are subsequently degraded 54. Although ligand-stimulated EGFR vesicular trafficking has been extensively studied, little is known about the role of vesicular trafficking in suppressing spontaneous EGFR activation and regulating its signalling response. A previous report also showed that without binding to growth factors, spontaneously active EGFRs on the cell surface are also endocytosed into vesicles. However, unlike ligand-activated EGFRs, the spontaneously active receptors are recycled back to the membrane 55. During recycling, their activity is also quenched by encountering phosphatases, and these receptors are not active when they return back to the cell surface. Interestingly, our results showed that KD of BCAP31 increased the recycling of EGFR to the membrane and the loss of resident EGFR in endocytic vesicles, decreasing overall membrane EGFR expression and autophosphorylation.

Via observations of the interaction of BCAP31 with EGFR and its intracellular distribution, we found that although BCAP31 is mainly anchored to the ER, it co-localizes with EGFR throughout the whole endocardium system (Figure 5A-C). Our data further validated the critical role of BCAP31 in regulating EGFR recycling and downstream signalling. Interestingly, protein tyrosine phosphatases (PTPs), such as TP1B and TCPTP 56, 57, which act on EGFR with high catalytic performance, are segregated from the plasma membrane by association with the cytoplasmic membrane leaflet of the ER where BCAP31 resides. PTPs can dephosphorylate endocytosed ligand-bound EGFR. Additionally, we found that BCAP31 participates in the distribution of active EGFR and that the regulatory roles of BCAP31 in EGFR intracellular recycling and cancer development are RAB11-dependent. The well-known GTPase RAB11 is localized in the endosomal recycling compartment and can regulate EGFR recycling. Our data showed that overexpression of the ER-anchored protein BCAP31 affects the interaction between RAB11 and EGFR. Our results further suggest that the ER may provide a platform to initiate the formation of the EGFR-RAB11 complex and facilitate EGFR recycling, while BCAP31 is an independent inhibitory factor. Thus, downregulation of BCAP31 expression or blockade of its anchor at the ER may intervene in EGFR ligand-independent signalling, which is a potential therapeutic target in TNBC. Interestingly, recent papers have also shown that BCAP31 is expressed on the surfaces of human pluripotent stem cells and some cancer cells and regulates stemness by interacting with EpCAM 58, 59; therefore, the possibility that BCAP31 may exert other regulatory effects on EGFR recycling cannot be excluded and warrants further investigation.

In summary, the results of our study demonstrate that BCAP31 is a key player in TNBC development and that it participates in the spatial re-distribution, signalling kinetics, and recycling of EGFR. BCAP31 functions as a specific adaptor, inhibiting EGFR auto-recycling and sustaining EGFR auto-phosphorylation, which allows the prolonged activation of downstream AKT signalling. BCAP31 is a potential prognostic marker and therapeutic target in TNBC, and designing inhibitors targeting BCAP31 expression may be a promising approach to control TNBC, especially in combination with EGFR inhibitors.

## Supplementary Material

Supplementary figures and tables.

## Figures and Tables

**Table 1:** Comparison of clinicopathological profiles between low and high BCAP31 expression in BC patients. (n = 186 patients)*

	BCAP31 expression				
	High (N = 87)		Low (N = 99)		
Clinical Valuable	No. of patients	%	No. of patients	%	p Value
Age (years), median (range)^a^	50 (33-76)		51 (32-80)		0.9849
Age, years^b^					
≤50	44	50.57	49	49.49	0.8832
>50	43	49.43	50	50.51	
Grade^b^					
High	2	2.30	2	2.02	0.8607
Intermediate	54	62.07	58	58.59	
Low	20	22.99	22	22.22	
Unknown	11	12.64	17	17.17	
pTNM stage^b#^					
I	19	21.84	39	37.37	0.0756
II	50	57.47	48	48.48	
III	18	20.69	13	13.13	
NA	0	0.00	1	0.54	
Tumor size (cm)^b^					
≤2	36	41.38	49	48.48	0.3698
2~5	46	52.87	43	43.43	
>5	5	5.75	6	6.06	
Unknown	0	0.00	2	2.02	
LN status^b^					
Negative	50	57.47	64	64.65	0.3534
Positive	37	42.53	34	34.34	
Unknown	0	0.00	1	1.01	
Molecular subtype^b^					
Luminal A	11	12.64	30	30.30	0.0000
Luminal B	14	16.09	29	29.29	
HER2+	5	5.75	20	20.20	
TNBC	57	65.52	20	20.20	

* LN, lymph node; TNBC, triple negative breast cancer; pTNM, pathologic tumor, lymph node, metastasis classification. ^#^ pTNM stage IV was not included in this cohort of patients. ^a^ Student's t test. ^b^ P values based on Pearson's Chi-square test for categorical (counts, percentage) variables.
