# Supplementary material for: BCAP31 drives TNBC development by modulating ligand-independent EGFR trafficking and spontaneous EGFR phosphorylation
Source: Theranostics. 2019 Aug 21;9(22):6468–84. doi: 10.7150/thno.35383 (PMC6771250; doi:10.7150/thno.35383)
Supplement: Supplementary file 1 — Supplementary figures and tables. [file thnov09p6468s1.pdf]

Supplementary Figures

Figure S1

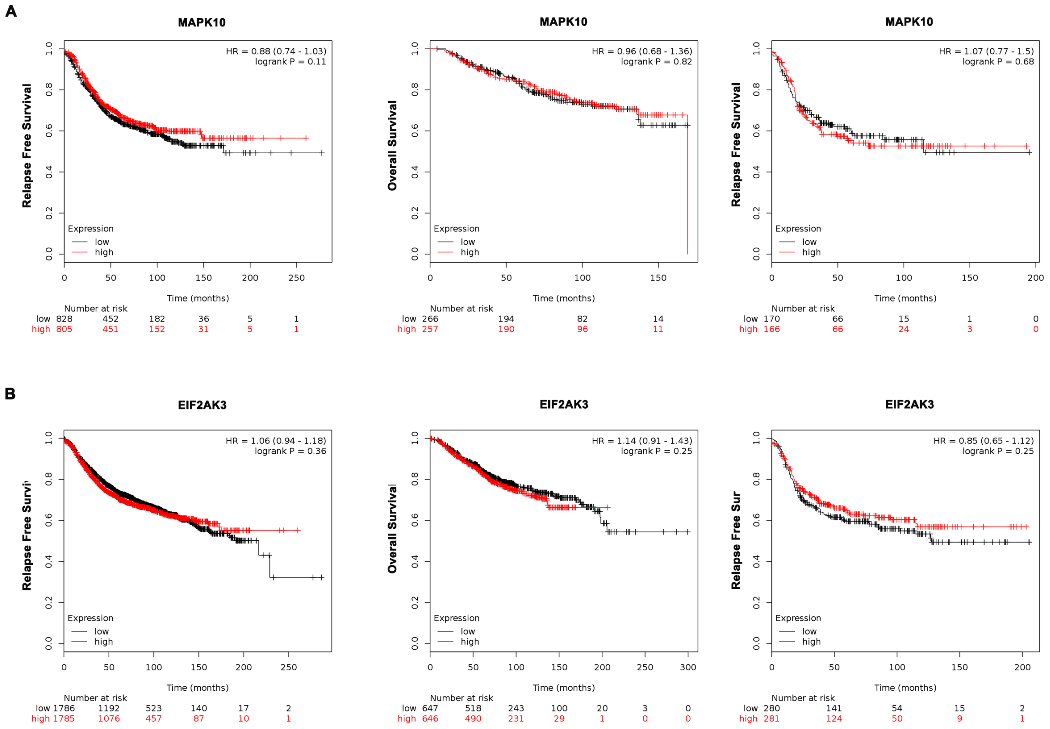

Figure S1. Kaplan–Meier survival curves of MAPK10 (A) and EIF2AK3 (B). Data showed no difference in RFS and OS in breast cancer (left two panel) or in basal-like breast cancer (right panel) with different gene expression levels.

Figure S2

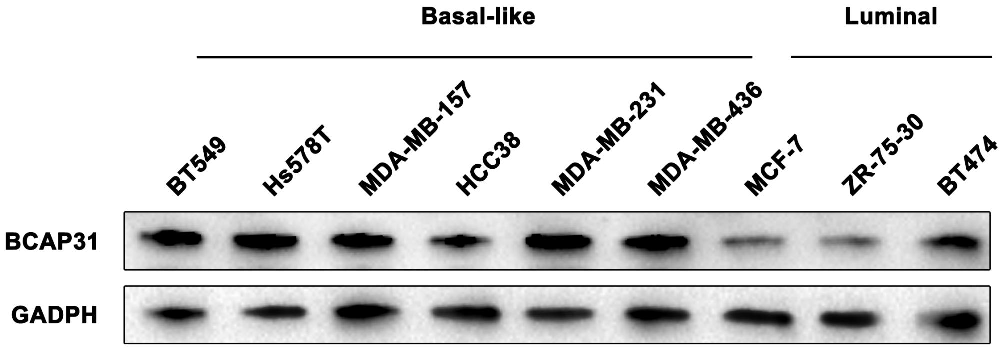

Figure S2. Expression of BCAP31 in different breast cancer cell lines. Basal-like and luminal-like breast cancer cell lines were lysed and immunoblotted with the indicated antibodies

Figure S3

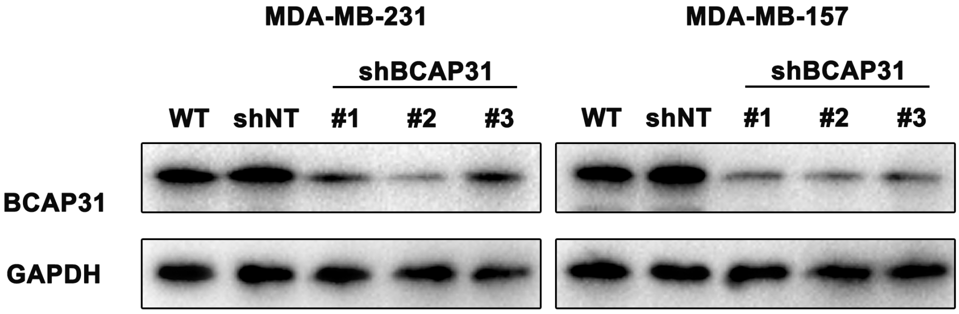

Figure S3. ShRNA-mediated BCAP31 down-regulation. Confirmation of BCAP31 knockdown in TNBC cell lines. Cells with different treatment were lysed, subjected to SDS-PAGE, and immunoblotted with the indicated antibodies.

Figure S4

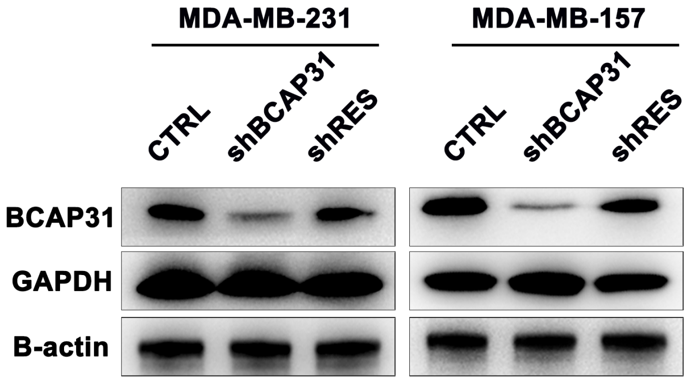

Figure S4. Expression of Housekeeping Genes in cancer cells with BCAP31 down-regulation. Confirmation of the expression of Housekeeping Genes in BCAP31 down-regulated TNBC cell lines. Cells with different treatment were lysed, subjected to SDS-PAGE, and immunoblotted with the indicated antibodies.

Figure S5

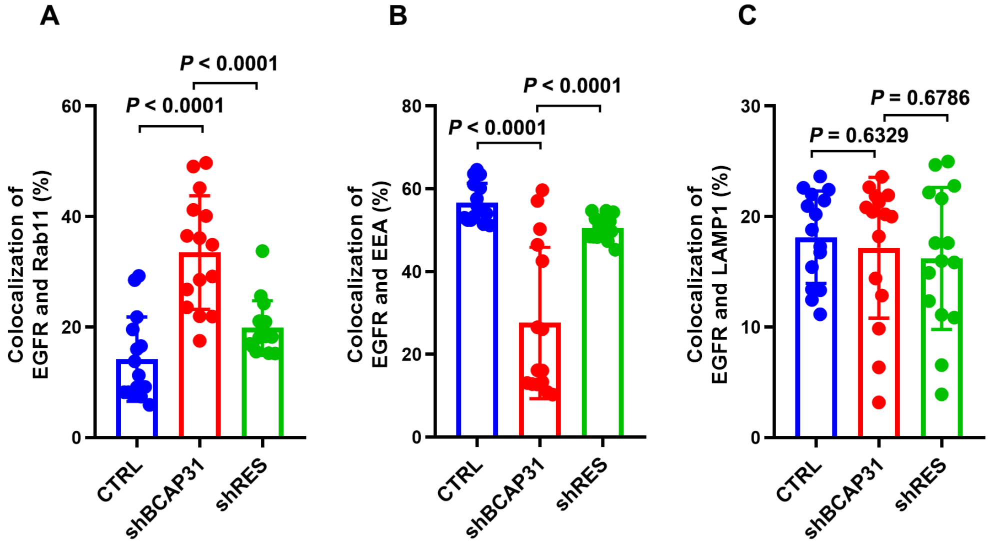

Figure S5. Colocalization of EGFR and different cellular markers. The colocalization percentage (%) of EGFR and **RAB11 (A), EEA (B), and LAMP1 (C)** are presented as mean ± SEM

Table S1. Univariate and multivariate analysis of factors associated with overall survival and disease-free survival*

	OS				DFS			
	Univariate	Multivariate			Univariate	Multivariate		
Clinical Valuable	p Value	HR	95% CI	p Value	p Value	HR	95% CI	p Value
Age, years								
⩽50	Ref.				Ref.			/
＞50	0.4701			/	0.8772			/
Grade								
High	NA			/	NA			
Intermediate	0.9429			/	0.9468			
Low	0.2219			/	0.4232			/
Unknown	Ref.			/	Ref.			/
pTNM stage[#]								
I	Ref.			Ref.	Ref.			/
II	0.1042	1.2957	0.1046-16.0574	0.8401	0.4278			/
III	0.0029	2.7838	0.1904-40.7006	0.4544	0.0223			/
Unknown	NA			NA	NA			/
Tumor size (cm)								
≤2	Ref.			Ref.	Ref.			Ref.
2~5	0.5299	1.6095	0.4682-5.5329	0.4500	0.8310	1.0536	0.5473-2.0282	0.8759
＞5	0.0001	6.3936	1.6588-24.6438	0.0070	0.0035	3.5497	1.3804-9.1279	0.0086

Unknown	NA			NA	NA			NA
LN status								
Negative	Ref.			Ref.	Ref.			Ref.
Positive	0.0004	4.1136	0.9966-16.9795	0.0506	0.0071	2.0716	1.1179-3.8390	0.0207
Unknown	NA			NA	NA			NA
Molecular subtype								
Luminal A	Ref.			/	Ref.			/
Luminal B	0.4555			/	0.8853			/
HER2+	0.5876			/	0.7242			/
TNBC	0.2725			/	0.8822			/
BCAP31 expression								
High	Ref.			Ref.	Ref.			Ref.
Low	0.0050	0.2026	0.0660-0.6219	0.0053	0.0446	0.5170	0.2781-0.9611	0.0370

* OS, overall survival; DFS, disease-free survival; HR, hazard ratio; 95% CI, 95% confidence interval; Ref., reference group; LN, lymph node; TNBC, triple negative breast cancer; pTNM, pathologic tumor, lymph node, metastasis classification.; NA, not applicable; /, not adopted.

# pTNM stage IV was not included in this cohort of patients.

Cox proportional hazard regression analysis for overall survival and disease-free survival were used (two-sided). Covariates used for multivariable-adjusted analysis for OS: pTNM stage, Tumor size, LN status, BCAP31 expression. Covariates used for multivariable-adjusted analysis for DFS: Tumor size, LN status, BCAP31 expression.

Table S2 Summary of mass spectrometry(MS) analysis of BCAP31 interactors associated with EGFR signaling and membrane endocytosis and traffic.

Band (kD)	Indentified proteins	Description	Accession	Function and process
28	BCAP31	B-cell receptor-associated protein 31	P51572	Functions as a chaperone protein. Is one of the most abundant endoplasmic reticulum (ER) proteins. Plays a role in the export of secreted proteins in the ER, the recognition of abnormally folded protein and their targeting to the ER associated-degradation (ERAD). Also serves as a cargo receptor for the export of transmembrane proteins. May be involved in CASP8-mediated apoptosis
44.9	LAMP2	Lysosome-associated membrane glycoprotein 2	P13473	Plays an important role in chaperone-mediated autophagy, a process that mediates lysosomal degradation of proteins in response to various stresses and as part of the normal turnover of proteins with a long biological half-live (PubMed:8662539, PubMed:11082038, PubMed:18644871, PubMed:24880125, PubMed:27628032). Functions by binding target proteins, such as GAPDH and MLLT11, and targeting them for lysosomal degradation (PubMed:8662539, PubMed:11082038, PubMed:18644871, PubMed:24880125). Plays a role in lysosomal protein degradation in response to starvation (By similarity). Required for the fusion of autophagosomes with lysosomes during autophagy (PubMed:27628032). Cells that lack LAMP2 express normal levels of VAMP8, but fail to accumulate STX17 on autophagosomes, which is the most likely explanation for the lack of fusion between autophagosomes and lysosomes (PubMed:27628032). Required for normal degradation of the contents of autophagosomes (PubMed:27628032). Required for efficient MHCII-mediated presentation of exogenous antigens via its function in lysosomal protein degradation; antigenic peptides generated by proteases in the endosomal/lysosomal compartment are captured by nascent MHCII subunits

				(PubMed:20518820). Is not required for efficient MHCII-mediated presentation of endogenous antigens (PubMed:20518820)
44.9	LAMP1	Lysosome-associated membrane glycoprotein 1	P11279	Presents carbohydrate ligands to selectins. Also implicated in tumor cell metastasis. Acts as a receptor for Lassa virus protein.
99.8	CDH2	Cadherin-2	P19022	Cadherins are calcium-dependent cell adhesion proteins. They preferentially interact with themselves in a homophilic manner in connecting cells; cadherins may thus contribute to the sorting of heterogeneous cell types. Acts as a regulator of neural stem cells quiescence by mediating anchorage of neural stem cells to ependymocytes in the adult subependymal zone: upon cleavage by MMP24, CDH2-mediated anchorage is affected, leading to modulate neural stem cell quiescence. CDH2 may be involved in neuronal recognition mechanism. In hippocampal neurons, may regulate dendritic spine density (By similarity)
136.3	WASH7	WASH complex subunit 4	Q2M389	Acts at least in part as component of the WASH core complex whose assembly at the surface of endosomes seems to inhibit WASH nucleation-promoting factor (NPF) activity in recruiting and activating the Arp2/3 complex to induce actin polymerization, and which is involved in the regulation of the fission of tubules that serve as transport intermediates during endosome sorting (PubMed:19922875, PubMed:20498093)
	RAB5C	Ras-related protein Rab-5C	RAB5C	Protein transport. Probably involved in vesicular traffic
20.4	RAB14	Ras-related protein Rab-14	P61106	Involved in membrane trafficking between the Golgi complex and endosomes during early embryonic development. Regulates the Golgi to endosome transport of FGFR-containing vesicles during early development, a key process for developing

				basement membrane and epiblast and primitive endoderm lineages during early postimplantation development. May act by modulating the kinesin KIF16B-cargo association to endosomes (By similarity). Regulates, together with its guanine nucleotide exchange factor DENND6A, the specific endocytic transport of ADAM10, N-cadherin/CDH2 shedding and cell-cell adhesion.
23.5	RAB7A	Ras-related protein Rab-7a	P51149	Key regulator in endo-lysosomal trafficking. Governs early-to-late endosomal maturation, microtubule minus-end as well as plus-end directed endosomal migration and positioning, and endosome-lysosome transport through different protein-protein interaction cascades. Plays a central role, not only in endosomal traffic, but also in many other cellular and physiological events, such as growth-factor-mediated cell signaling, nutrient-transportor mediated nutrient uptake, neurotrophin transport in the axons of neurons and lipid metabolism. Also involved in regulation of some specialized endosomal membrane trafficking, such as maturation of melanosomes, pathogen-induced phagosomes (or vacuoles) and autophagosomes. Plays a role in the maturation and acidification of phagosomes that engulf pathogens, such as S.aureus and M.tuberculosis. Plays a role in the fusion of phagosomes with lysosomes. Plays important roles in microbial pathogen infection and survival, as well as in participating in the life cycle of viruses. Microbial pathogens possess survival strategies governed by RAB7A, sometimes by employing RAB7A function (e.g. Salmonella) and sometimes by excluding RAB7A function (e.g. Mycobacterium). In concert with RAC1, plays a role in regulating the formation of RBs (ruffled borders) in osteoclasts. Controls the endosomal trafficking and neurite outgrowth signaling of NTRK1/TRKA (PubMed:11179213,

				PubMed:12944476, PubMed:14617358, PubMed:20028791, PubMed:21255211). Regulates the endocytic trafficking of the EGF-EGFR complex by regulating its lysosomal degradation. Involved in the ADRB2-stimulated lipolysis through lipophagy, a cytosolic lipase-independent autophagic pathway (By similarity). Required for the exosomal release of SDCBP, CD63 and syndecan (PubMed:22660413)
23.6	RAB6A	Ras-related protein Rab-6A	P20340	Protein transport. Regulator of membrane traffic from the Golgi apparatus towards the endoplasmic reticulum (ER). Has a low GTPase activity. Involved in COPI-independent retrograde transport from the Golgi to the ER (PubMed:25962623)
23.4	RAB6B	Ras-related protein Rab-6B	Q9NRW1	Seems to have a role in retrograde membrane traffic at the level of the Golgi complex. May function in retrograde transport in neuronal cells.
17.7	RAB11A	Ras-related protein Rab-11A	P62491	The small GTPases Rab are key regulators of intracellular membrane trafficking, from the formation of transport vesicles to their fusion with membranes. Rabs cycle between an inactive GDP-bound form and an active GTP-bound form that is able to recruit to membranes different set of downstream effectors directly responsible for vesicle formation, movement, tethering and fusion. That Rab regulates endocytic recycling. Acts as a major regulator of membrane delivery during cytokinesis. Together with MYO5B and RAB8A participates in epithelial cell polarization. Together with RAB3IP, RAB8A, the exocyst complex, PARD3, PRKCI, ANXA2, CDC42 and DNMBP promotes transcytosis of PODXL to the apical membrane initiation sites (AMIS), apical surface formation and lumenogenesis. Together with MYO5B participates in CFTR trafficking to the plasma membrane and TF (Transferrin) recycling in nonpolarized cells. Required in a complex with MYO5B and

				RAB11FIP2 for the transport of NPC1L1 to the plasma membrane. Participates in the sorting and basolateral transport of CDH1 from the Golgi apparatus to the plasma membrane. Regulates the recycling of FCGRT (receptor of Fc region of monomeric Ig G) to basolateral membranes. May also play a role in melanosome transport and release from melanocytes.
20.5	CAV1	Caveolin-1	Q03135	May act as a scaffolding protein within caveolar membranes. Interacts directly with G-protein alpha subunits and can functionally regulate their activity (By similarity). Involved in the costimulatory signal essential for T-cell receptor (TCR)-mediated T-cell activation. Its binding to DPP4 induces T-cell proliferation and NF-kappa-B activation in a T-cell receptor/CD3-dependent manner. Recruits CTNNB1 to caveolar membranes and may regulate CTNNB1-mediated signaling through the Wnt pathway. Negatively regulates TGFB1-mediated activation of SMAD2/3 by mediating the internalization of TGFBR1 from membrane rafts leading to its subsequent degradation (PubMed:25893292). Mediates the recruitment of CAVIN proteins (CAVIN1/2/3/4) to the caveolae (PubMed:19262564)
27.9	VAPA	Vesicle-associated membrane protein-associated protein A	Q9P0L0	Binds to OSBPL3, which mediates recruitment of VAPA to plasma membrane sites (PubMed:25447204). The ORP3-VAPA complex stimulates RRAS signaling which in turn attenuates integrin beta-1 (ITGB1) activation at the cell surface (PubMed:25447204). With OSBPL3, may regulate ER morphology (PubMed:16143324). May play a role in vesicle trafficking (PubMed:11511104, PubMed:19289470)
20.5	VPS29	Vacuolar protein sorting-	Q9UBQ0	Acts as component of the retromer cargo-selective complex (CSC). The CSC is believed to be the core functional component

		associated protein 29		of retromer or respective retromer complex variants acting to prevent missorting of selected transmembrane cargo proteins into the lysosomal degradation pathway. The recruitment of the CSC to the endosomal membrane involves RAB7A and SNX3. The SNX-BAR retromer mediates retrograde transport of cargo proteins from endosomes to the trans-Golgi network (TGN) and is involved in endosome-to-plasma membrane transport for cargo protein recycling. The SNX3-retromer mediates the retrograde endosome-to-TGN transport of WLS distinct from the SNX-BAR retromer pathway. The SNX27-retromer is believed to be involved in endosome-to-plasma membrane trafficking and recycling of a broad spectrum of cargo proteins. The CSC seems to act as recruitment hub for other proteins, such as the WASH complex and TBC1D5. Required to regulate transcytosis of the polymeric immunoglobulin receptor (pIgR-pIgA) (Probable). Involved in GLUT1 endosome-to-plasma membrane trafficking; the function is dependent of association with ANKRD27 (PubMed:24856514)
35.6	SNX1	Sorting nexin-1	Q13596	nvolved in several stages of intracellular trafficking. Interacts with membranes containing phosphatidylinositol 3-phosphate (PtdIns(3P)) or phosphatidylinositol 3,5-bisphosphate (PtdIns(3,5)P2) (PubMed:12198132). Acts in part as component of the retromer membrane-deforming SNX-BAR subcomplex. The SNX-BAR retromer mediates retrograde transport of cargo proteins from endosomes to the trans-Golgi network (TGN) and is involved in endosome-to-plasma membrane transport for cargo protein recycling. The SNX-BAR subcomplex functions to deform the donor membrane into a tubular profile called endosome-to-TGN transport carrier (ETC) (Probable). Can sense membrane curvature and has in vitro vesicle-to-membrane remodeling activity

				(PubMed:19816406, PubMed:23085988). Involved in retrograde endosome-to-TGN transport of lysosomal enzyme receptors (IGF2R, M6PR and SORT1) and Shiginella dysenteria toxin stxB. Plays a role in targeting ligand-activated EGFR to the lysosomes for degradation after endocytosis from the cell surface and release from the Golgi (PubMed:12198132, PubMed:15498486, PubMed:17550970, PubMed:17101778, PubMed:18088323, PubMed:21040701). Involvement in retromer-independent endocytic trafficking of P2RY1 and lysosomal degradation of protease-activated receptor-1/F2R (PubMed:16407403, PubMed:20070609). Promotes KALRN- and RHOG-dependent but retromer-independent membrane remodeling such as lamellipodium formation; the function is dependent on GEF activity of KALRN (PubMed:20604901). Required for endocytosis of DRD5 upon agonist stimulation but not for basal receptor trafficking (PubMed:23152498)
134.3	EGFR	Epidermal growth factor receptor	P00533	Receptor tyrosine kinase binding ligands of the EGF family and activating several signaling cascades to convert extracellular cues into appropriate cellular responses (PubMed:2790960, PubMed:10805725, PubMed:27153536). Known ligands include EGF, TGFA/TGF-alpha, AREG, epigen/EPGN, BTC/betacellulin, epiregulin/EREG and HBEGF/heparin-binding EGF (PubMed:2790960, PubMed:7679104, PubMed:8144591, PubMed:9419975, PubMed:15611079, PubMed:12297049, PubMed:27153536, PubMed:20837704). Ligand binding triggers receptor homo- and/or heterodimerization and autophosphorylation on key cytoplasmic residues. The phosphorylated receptor recruits adapter proteins like GRB2 which in turn activates complex downstream signaling cascades. Activates at least 4 major downstream signaling cascades including the

				RAS-RAF-MEK-ERK, PI3 kinase-AKT, PLCgamma-PKC and STATs modules (PubMed:27153536). May also activate the NF-kappa-B signaling cascade (PubMed:11116146). Also directly phosphorylates other proteins like RGS16, activating its GTPase activity and probably coupling the EGF receptor signaling to the G protein-coupled receptor signaling (PubMed:11602604). Also phosphorylates MUC1 and increases its interaction with SRC and CTNNB1/beta-catenin (PubMed:11483589). Plays a role in enhancing learning and memory performance
50.6	GDI2	Rab GDP dissociation inhibitor beta	P50395	Regulates the GDP/GTP exchange reaction of most Rab proteins by inhibiting the dissociation of GDP from them, and the subsequent binding of GTP to them.
53.6	VIM	Vimentin	P08670	Vimentins are class-III intermediate filaments found in various non-epithelial cells, especially mesenchymal cells. Vimentin is attached to the nucleus, endoplasmic reticulum, and mitochondria, either laterally or terminally.
